# Hot Spots of Site-Specific Integration into the *Sinorhizobium meliloti* Chromosome

**DOI:** 10.3390/ijms251910421

**Published:** 2024-09-27

**Authors:** Maria E. Vladimirova, Marina L. Roumiantseva, Alla S. Saksaganskaia, Victoria S. Muntyan, Sergey P. Gaponov, Alessio Mengoni

**Affiliations:** 1 Laboratory of Genetics and Selection of Microorganisms, Federal State Budget Scientific Institution All-Russia Research Institute for Agricultural Microbiology (FSBSI ARRIAM), 196608 Saint Petersburg, Russia; mariiacherkasova@arriam.ru (M.E.V.); allasaksaganskaya@arriam.ru (A.S.S.); vucovar@yandex.ru (V.S.M.); 2 Novikov Labs, 420033 Kazan, Russia; bioisergey@gmail.com; 3 Department of Biology, University of Florence, 50019 Sesto Fiorentino, Italy; alessio.mengoni@unifi.it

**Keywords:** rhizobiophages, prophages, genomic islands, site-specific integration, hot spots, integrases, tRNA genes, *oriC/terC*, co-evolution of bacteria and phages, *Sinorhizobium meliloti*

## Abstract

The diversity of phage-related sequences (PRSs) and their site-specific integration into the genomes of nonpathogenic, agriculturally valuable, nitrogen-fixing root nodule bacteria, such as *Sinorhizobium meliloti*, were evaluated in this study. A total of 314 PRSs, ranging in size from 3.24 kb to 88.98 kb, were identified in the genomes of 27 *S. meliloti* strains. The amount of genetic information foreign to *S. meliloti* accumulated in all identified PRSs was 6.30 Mb. However, more than 53% of this information was contained in prophages (Phs) and genomic islands (GIs) integrated into genes encoding tRNAs (tRNA genes) located on the chromosomes of the rhizobial strains studied. It was found that phiLM21-like Phs were predominantly abundant in the genomes of *S. meliloti* strains of distant geographical origin, whereas RR1-A- and 16-3-like Phs were much less common. In addition, GIs predominantly contained fragments of phages infecting bacteria of distant taxa, while rhizobiophage-like sequences were unique. A site-specific integration analysis revealed that not all tRNA genes in *S. meliloti* are integration sites, but among those in which integration occurred, there were “hot spots” of integration into which either Phs or GIs were predominantly inserted. For the first time, it is shown that at these integration “hot spots”, not only is the homology of *attP* and *attB* strictly preserved, but integrases in PRSs similar to those of phages infecting the Proteobacteria genera *Azospirillum* or *Pseudomonas* are also present. The data presented greatly expand the understanding of the fate of phage-related sequences in host bacterial genomes and also raise new questions about the role of phages in bacterial–phage coevolution.

## 1. Introduction

The successful ecological adaptation of bacteria may depend on the ability to expand the bacterial genome with genetic material, which the cell can obtain through horizontal gene transfer processes, including transduction [1,2,3]. Phage DNA is integrated into the bacterial genome through site-specific recombination and can remain there for a long time as part of the replicon that was targeted for integration; alternatively, if its mobility is lost, the “grounding” of the phage DNA sequence in the host bacterial genome occurs [4,5]. In bacteria, phage-related sequences are present in significant numbers and can account for up to 10–20% of the genome [6,7,8]. For example, up to 18 and 6 of such sequences are found in *E. coli* and *Desulfovibrio vulgaris* strains, respectively, and their presence in a genome does not depend on the genetic background [9,10,11].

Once integrated into the host bacterial genome, temperate phages can persist for a long time as prophages that evolve further through intragenomic recombination [12]. Prophages can be subdivided into three groups as follows: (i) intact prophages (int-Phs), which are sequences capable of excision and existence as part of a viral particle; (ii) incomplete prophages (inc-Phs), which are sequences that have lost some or all structural genes responsible for excision and/or virulence; and (iii) questionable prophages (q-Phs), which cannot be classified as intact or incomplete [13,14]. All of these sequences (int-Ph, q-Ph, and inc-Ph) have different levels of integrity and can be grouped together as “phage-related sequences” (PRSs). Genomic islands (GIs) containing not only genes of phage origin but also a significant number of genes of bacterial origin can also belong to this group [15]. This is consistent with the hypothesis that genomic islands originated from prophages or plasmids [4,5].

The integration or loss of PRSs may contribute to intraspecific variability in bacterial strains, along with mutations and genomic rearrangements [16,17,18,19,20,21]. The presence of genes or gene blocks in phage-related sequences can confer new, previously absent properties to the host bacterium (lysogenic conversion), which has been studied mainly in pathogenic bacteria and single strains of nonpathogenic bacteria. For example, changes in bacterial properties such as pathogenicity [4,22,23,24], antibiotic resistance [25], immunity [26,27,28], symbiotic activity [29,30,31], and viability and adaptability to environmental conditions [5,32,33,34,35,36] have been observed. However, the expression level of the introduced genetic material depends on the intact genomic background of the bacterium, which may or may not support the expression or function of the recruited genes, consistent with [37]. Prophages have been shown to affect the activity of recombination processes in the host genome [5,38,39,40], which may play a significant role in rapid bacterial evolution. In addition, the presence of prophages can influence the ecology of microbiocenosis. Thus, phage infection results in the immunization of bacterial hosts and protects them from superinfection by other closely related or unrelated phages [41]. All of these effects contribute to the viability of potential host bacteria, making the study of antiphage defense systems in the host bacterial genome all the more relevant [5,12,42,43,44,45].

**Figure 1:**
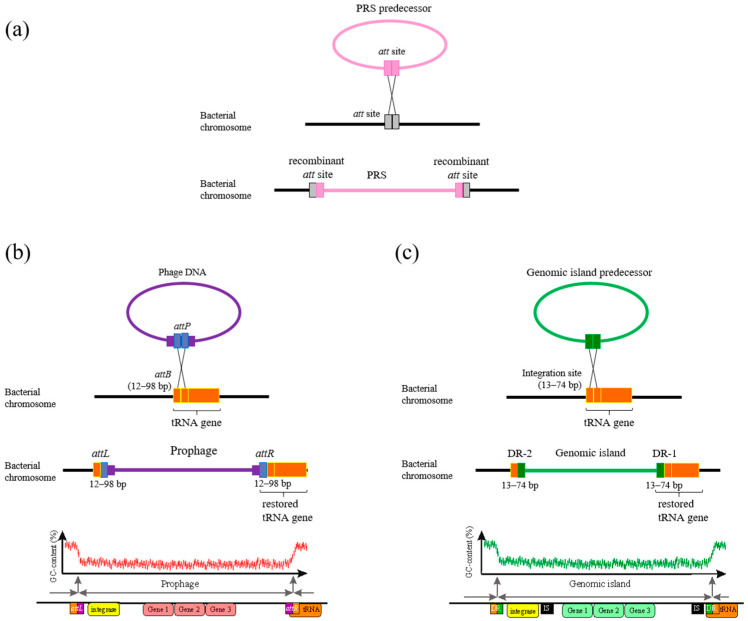
General scheme of PRS integration into bacteria genome (**a**) adopted from [5]. PRS predecessor—the PRS before integration into a bacterial chromosome. *att* site—attachment site for PRS integration. Scheme of integration of a phage (**b**) and a genomic island (**c**) into the tRNA gene, designed according to the data obtained in this work for *S. meliloti* strains and adopted from [5,48,49]. *attP*, *attB*—sites for phage integration (see text); *attL*, *attR*—recombinant sites (see text); DR-1 and -2—short direct repeat sequences flanking the GI (see text).

The integration of a PRS into the host bacterial genome occurs through site-specific recombination with the participation of enzymes (Figure 1a), which are conserved site-specific tyrosine and serine recombinases belonging to two unrelated families. These recombinases are widely distributed in bacterial and viral genomes and are often referred to as “phage-like integrases”, responsible for the mobility of mobile genetic elements of different types [46,47].

In most cases, the attachment site (*attB*) of a tyrosine integrase in the bacterial genome corresponds to a region of a gene encoding transfer or transfer–messenger RNA (tRNA or tmRNA gene, respectively) and that fact has the biological importance of preserving the integrity and function of essential tRNA genes after PRS integration (Figure 1) [50,51,52,53,54]. Attachment sites are usually short 15–30 bp sequences that typically correspond to the sequence at the 3′ end of the tRNA gene and, in some cases, *attB* sequences may extend beyond the gene boundary. However, it has been suggested that the local genetic context of the *attB* site may not be crucial for PRS integration [10].

The attachment site of a PRS (*attP*) undergoes recombination with an *attB* site to generate the recombinant sites *attL* and *attR* (left and right sites), which are direct repeats flanking the PRS (DR) integrated into the bacterial genome (Figure 1a). The *attP* site in the phage genome is much more complex and longer than the *attB* site and can bind to recombination proteins [5,48,49].

The fact that the site-specific integration of phage sequences occurs precisely in tRNA genes may be due to a number of factors, including the conservation of evolutionarily ancient genes and their small size. This is thought to be responsible for minimizing the size of the host bacterial DNA sequences used in *attP*, which is necessary to restore gene function in the case of phage sequence integration (Figure 1) [50]. For some species of bacteria, not all tRNA genes are integration sites, and there may be other sites in the genome where PRS integration is more active [55,56].

Recently, it was shown that the integration of PRSs into the chromosome of *Alphaproteobacteria* of the *Rhodobacteraceae* family depended on the structural organization of the chromosome and, above all, on the location of *oriC* (replication origin) and *terC* (replication terminus) [56,57]. The sequence of the *dnaA*-encoding gene is among the conserved, taxonomically significant markers used to determine *oriC* and thus is the starting point for chromosomal gene coordinates [58,59]. The location of *terC* is less well studied, and its location is not universal; therefore, techniques based on the analysis of chromosome nucleotide composition asymmetry are used to search for potential *terC* sequences. Both of these regions have been described for a limited number of bacteria [60].

Information on the integration sites and origins of phage sequences, as well as on the structural organization of chromosomes, is mainly known for pathogenic bacterial strains including strains of *Pseudomonas*, *Staphylococcus*, *Streptococcus*, *Bacillus*, and *E. coli* [5,24,25,27,32]. For nonpathogenic species such as *Sinorhizobium meliloti*, which are nitrogen-fixing symbionts of legume crops, such data are almost nonexistent.

*S. meliloti* are agriculturally valuable alfalfa symbionts and typical representatives of the soil microbiome. These bacteria have a multicomponent genome that includes a chromosome and two megaplasmids, and they often contain nonsymbiotic plasmids that vary greatly in number and size. There are data on the structure of the chromosome of the model strain *S. meliloti* 1021 (Rm1021), as well as on the locations of extended chromosomal genomic islands, which are presumably of phage origin [15,61,62]. The predicted functions of the ORFs of *S. meliloti* 1021 genomic islands showed that these sequences are similar to the ORFs of bacteria belonging to at least 10 different classes in six different phyla, which, together, suggests the active participation of GIs in horizontal transfer processes [62]. GIs of similar localization, like GIs of Rm1021, were detected in almost half of the *S. meliloti* strains isolated from soil samples collected at the origin of plant diversity in the Northern Caucasus as well from an alfalfa introgressive hybridization center in Mugodzhary, NW Kazakhstan [20,63]. We found that some other *S. meliloti* isolates may have GIs whose localization differs from the above [21]. Thus, there is some understanding of the structure, location, and functional significance of genomic islands in *S. meliloti* genomes, but little is known about the origin, diversification, and specificity of GI integration into the rhizobial genome.

The aim of our study was to estimate the abundance of phage-related sequences (PRSs) in *S. meliloti* bacterial genomes based on analyses of full-genome data of strains independently sampled from geographically distant areas, according to the NCBI database (27 strains). For the first time, data on the site-specific integration of PRSs allowed us to identify integration hot spots on the *S. meliloti* chromosome. As a result of this study, the mechanisms underlying the integration of phage-originating sequences into bacterial genomes are better understood, allowing us to place a new focus on the evolution of bacterial–phage interactions.

## 2. Results

### 2.1. Phage-Related Sequences (PRSs)

Three hundred fourteen phage-related sequences (PRSs) were detected in the genomes of 27 strains of *Sinorhizobium meliloti* (Table 1), independently sampled from geographically distant areas (see Section 4 Materials and Methods). That is, on average, there could be 11 ± 1.1 PRSs per genome of *S. meliloti*, as predicted using PHASTER, PHASTEST and Islander (see Section 4 Materials and Methods). We identified seven inc-Phs in the genome of *S. meliloti* 1021—one on the chromosome, four on pSymA, and two on pSymB—in addition to the three extended genomic islands described previously [15].

PRSs, considered “foreign sequences” in rhizobial genomes, varied significantly in length, ranging from 3.24 to 88.98 kb. On average, there were 233 ± 25 kb of PRSs per genome (or 3.3 ± 0.3% of genome size), with minimum and maximum PRS lengths of 21.28 kb and 576.29 kb (0.8 to 7.9% of genome size), respectively. More than half of the PRSs were represented by inc-Phs (frequency of 0.52), and int-Phs and GIs occurred with equal frequencies (0.17). In addition, q-Phs were detected at a frequency of 0.14 (Table 1). The total length of all 314 PRSs was 6.30 Mb and included 7.4 thousand ORFs.

PRSs could be detected on any replicon in the rhizobial genome, and their distribution among replicons was not random (Χ^2^ = 22.0, P = 6.5 × 10^−5^). PRSs were found twice as frequently on chromosomes as on the megaplasmids pSymA and pSymB, on which they occurred at similar frequencies (0.42 and an average frequency of 0.21, respectively). PRSs were also found on 28 cryptic plasmids (frequency of 0.17), which were identified in the genomes of 16 of the 27 strains studied.

The analysis of the chromosome structure was a priority. Of the 314 PRSs, 132 PRSs with a total length of 4.09 Mb (i.e., 64.8% of the total amount of detected foreign DNA of phage origin) fell on the chromosomes of the studied strains (Table S1). On average, 152 ± 16 kb of foreign DNA was found per *S. meliloti* chromosome (the minimum and maximum average sizes were 12.2 and 307.9 kb, respectively). We found that 88 of the 132 PRSs were site-specifically integrated into tRNA genes. These 88 PRSs accounted for 53.5% of the detected foreign DNA of phage origin (Table S1). These PRSs were represented by 51 GIs (frequency of 0.58) and 37 prophages (Phs; Table 1).

Of the 37 prophages, 27 (frequency of 0.73) were similar to only three rhizobiophages (Table 2). The vast majority of these prophages were similar to the *Sinorhizobium* phage phiLM21 (frequency of 0.67); much less frequently, the phages were similar to *Rhizobium* phage 16-3 or phage RR1-A (with equal frequencies, 0.17). The other 10 of the 37 phages were similar to phages infecting *Mesorhizobium*, *Brucella*, *Sulfitobacter*, and *Enterobacteria* (Table 2), which are members of different orders including *Hyphomicrobiales* (*Mesorhizobium* and *Brucella*), *Rhodobacterales*, and *Enterobacteriales*, respectively.

We found it interesting that prophages similar to phages phiLM21 and RR1-A were only int-Ph (Table 2). In contrast, the following sequences similar to *Rhizobium* phage 16-3 were found with varying degrees of integrity: int-Phs, inc-Phs, and q-Phs (Table 2). The int-Phs were single sequences similar to phages infecting *Mesorhizobium*, *Brucella*, or *Sulfitobacter*, while sequences similar to phages infecting *Enterobacteria* occurred only as q-Phs (Table 2 and Table S2).

The analysis of the 51 GI sequences showed that 32 of them contained no sequences of phage origin, with the exception of the gene encoding integrase (see below). The analysis of the other 19 GIs (frequency of 0.37) showed that they included short sequences of phage origin annotated as inc-Phs and q-Phs, according to PHASTER and PHASTEST, but the extent of these sequences was less than 50% of the length of the genomic island.

Rhizobiophage-like sequences were in only 8 of the 19 GIs discussed above. These GIs contained single inc-Ph sequences similar to *Sinorhizobium* phage phiLM21, *Rhizobium* phage 16-3, and *Sinorhizobium* phage ort11, as well as inc-Phs similar to two *Sinorhizobium* phages, phiN3 and phiM7, and four q-Ph sequences similar to *Rhizobium* phage vB_RleM_PPF1. However, GIs may contain several distinct prophages at the same time. For example, the GI of strain AK21 has two inc-Phs simultaneously, one similar to *Sinorhizobium* phage phiLM21 and the other one similar to *Sulfitobacter* phage NYA_2014a. Similarity was found between phage sequences in GIs and a wide variety of phages infecting bacteria of distant taxa. For example, one of the q-Ph sequences similar to *Rhizobium* phage vB_RleM_PPF1 (strain T073) was similar to *Azospirillum* phage Cd and *Rhodobacter* phage RcapNL (Table S2).

The remaining 11 of the 19 GIs contained sequences similar to phages infecting *Brucella*, *Sulfitobacter*, and *Enterobacter* species, as well as sequences similar to three different Stx-encoding *Escherichia* phages (F451, C1717, SH2026) and *Ralstonia* phage RsoM1USA (USDA1106 strain). In addition, a q-Ph similar to a phage infecting *Pelagibacter* was present in one of these GIs (Table S2).

In summary, *S. meliloti* chromosomes contain prophages integrated into tRNA genes, which differ in their degree of structural integrity, and GIs with and without phage-like sequences. It was observed that int-Phs did not occur in the GIs: only inc-Phs and q-Phs were present in a ratio of 14:5. These sequences were mostly similar to phages infecting bacteria of taxonomically distant genera (frequency of 0.68). Interestingly, int-Phs were predominantly rhizobiophage-like and were integrated into tRNA genes, whereas rhizobiophage-like sequences were extremely rare in GIs (frequencies of 0.31 and 0.08, respectively).

### 2.2. Site-Specific Integration of PRSs

All 37 Phs and 51 GIs integrated into tRNA genes were found to be flanked by two short direct repeats (DRs) ranging in size from 12 to 98 bp, which are presumably recombinant sites named *attL* and *attR* (see Introduction) (Figure 1b,c). In this work, one of the paired DR sequences corresponding to the sequence at the 3′ or 5′ end of a tRNA gene was labeled *attR*, whereas the opposite DR sequence was always labeled *attL* (Figure 1b,c). *attR* matched the sequence at the 3′ end of the encoding gene in 77 of 88 PRSs (frequency of 0.88); in only nine cases did *attR* match the sequence at the 5’ end of the tRNA gene (frequency of 0.10) (Table S2). In all of these cases, the integrity of the tRNA genes was always preserved (Figure 1b,c).

Two cases were identified where *attR* corresponded to the sequence of the central part of the tRNA gene, resulting in a disruption of the tRNA gene structure. In these cases, the integration of int-Phs similar to *Mesorhizobium* phage vB_MloP_Lo5R7ANS led to the inactivation of the gene encoding tRNA^Leu^(UAG)_026_ (strains AK21 and RU11/001). However, in both cases, a gene functionally similar to tRNA was present in the int-Ph (see below).

The site-specific integration of PRSs occurred in only 25 out of all 52 tRNA genes on the chromosome. The sequence analysis of the genes encoding the corresponding tRNAs showed that their sequences were identical in each of the 27 strains studied with few exceptions. The sequences of three genes encoding tRNA^Arg^(ACG), tRNA^Leu^(CAA), and tRNA^Glu^(CTC) (strains 1021, AK170, and USDA1021, respectively) had SNPs. As a result, the average identity of these sequences was 98.7% (coverage 100%) relative to the corresponding sequences among all of the strains studied. In certain *S. meliloti* strains, the number of tRNA genes could be higher or lower than in the reference strain (1021) because of intrachromosomal rearrangements affecting the regions containing the tRNA genes (strains SM11, KH46, USDA1157, T073, B399). Thus, in strain B399, 60 tRNA genes were detected, which resulted from the duplication of a 223 kb fragment containing 8 tRNA genes (018-025) (Table 3). This is the highest number of tRNA genes among the *S. meliloti* genomes studied. The lowest number of tRNA genes, namely, 46 and 48, as in strains M270 and USDA1021, respectively, was due to the deletion of extended regions containing the corresponding genes (Table S2).

The locations of the above 25 tRNA genes were estimated relative to the structural organization of the chromosomes of the analyzed *S. meliloti* strains. The sequence of the *oriC* region was identical to that of the reference strain (477 bp) in 18 out of 27 cases, whereas in the remaining cases, SNPs, deletions, and/or insertions were detected (identity 97.91–99.79%; π = 4.28 × 10^−3^ (see Section 4 Materials and Methods)). Thus, the first nucleotide of the *oriC* region was taken as a reference point for the alignment of the chromosomes [65,66]. The *terC* region was defined for each studied chromosome, and its distance from *oriC* was 170° ± 0.6°. The *terC* region was determined for each chromosome examined, and its distance from *oriC* was 170° ± 0.6°. An exception in the position of *terC* relative to *oriC* was detected only in cases of genomic rearrangements affecting extended sequences. Thus, *terC* in strains M270, SM11, B399, and USDA1021 had coordinates 185°, 215°, 192°, and 156°, respectively. The length of the *terC* region was 8.26 kb, and the level of similarity to the reference was 99.8–100% in 10 out of 27 cases. In the remaining 17 cases, the size of the *terC* region ranged from 6.60 to 11.71 kb. The variability in the size of this region was due to the presence of one to four ORFs encoding transposases or hypothetical proteins.

**Figure 2:**
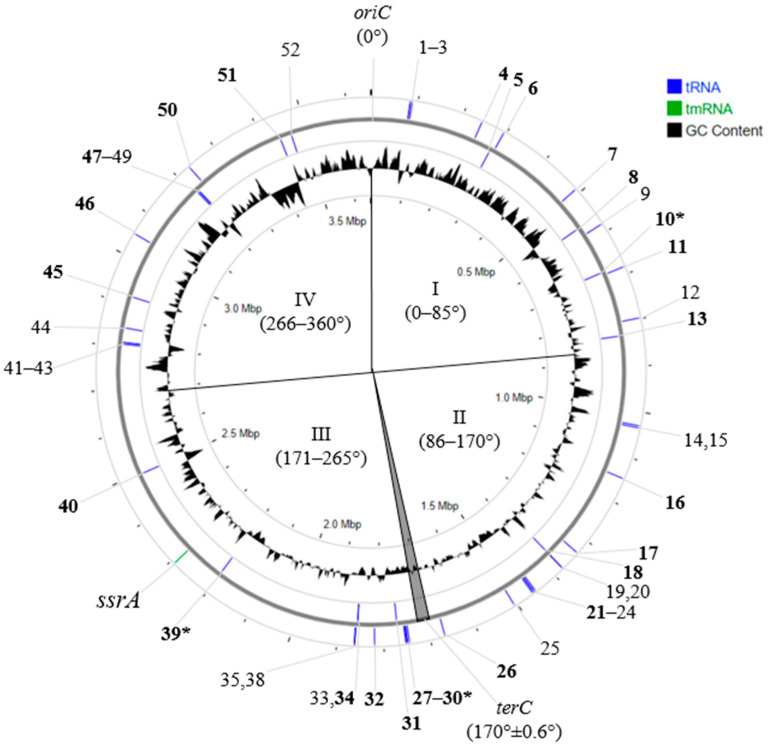
Localization of essential tRNA genes on *S. meliloti* 1021 chromosome. Designations: 1-52—ordinal numbers of tRNA genes (in the text 001-052); *oriC* and *terC*—origin and terminus of replication, respectively; I–IV—quarters of the chromosome, defined relative to *oriC* and *terC*; in bold—ordinal number of tRNA genes with integrated PRSs; *—“hot spots” of PRS integration (genes 030 and 039) and spot of preferential integration (gene 010) (see text).

Determining the *oriC*-*terC* axis made it possible to unify the locations of all 52 tRNA genes on the chromosomes and to show that the order of their locations is identical in all studied *S. meliloti* strains. An analysis of the locations of the above 25 tRNA genes into which PRS insertions occurred showed that they are relatively evenly distributed on the chromosome, with 13 and 12 of these tRNA genes on the plus and minus strands of DNA, respectively. Conversely, the distribution of all 52 tRNA genes between the plus and minus strands of the chromosome was significantly asymmetric (Χ^2^ = 15.8, P = 1.3 × 10^−3^) (Figure 2). This fact allowed us to unify the designation of genes encoding tRNAs and to give each gene its own ordinal number in the direction from *oriC* to *terC*, namely, 001-052 (further in the text, only the numbers of the corresponding genes will predominantly be used) (Figure 2; Table 3).

**Figure 3:**
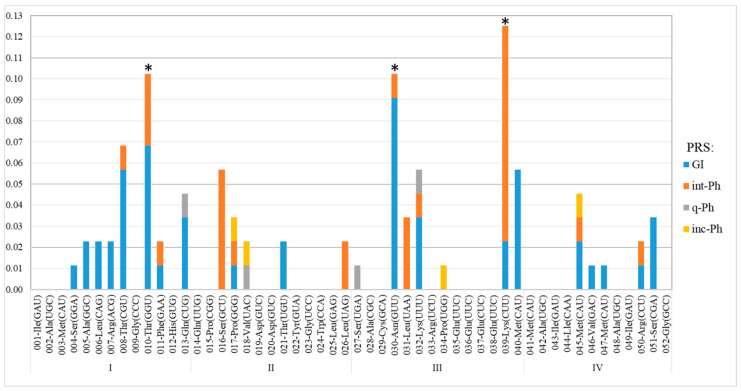
Frequencies of PRS integration in essential tRNA genes of *S. meliloti*. Designations: along the abscissa axis—the signature combines the ordinal number of tRNA genes, with a corresponding amino acid and anticodon following the dash (see Table 3); along the abscissa axis—frequency of occurrence of PRSs integrated into different tRNA genes; *—tRNA genes at “hot spots” of integration (genes 030 and 039) and spot of preferential integration (gene 010); I–IV—chromosome quarters defined relative to *oriC* and *terC* (see Figure 2).

The frequency of occurrence of PRSs integrated into any of the 25 tRNA genes was significantly different from a discrete uniform distribution, according to the Chi-square test (P = 1.97 × 10^−18^). We found that the average frequency of occurrence of integrated PRSs in the sequences of 16 out of the 25 tRNA genes was 0.02 ± 2 × 10^−3^; that in the other six tRNA genes (008, 013, 016, 032, 040, and 045) was almost threefold higher at 0.05 ± 3 × 10^−3^. The frequency of PRSs integrated into the sequences of the last 3 of the 25 tRNA genes (010, 030, and 039) was significantly higher, equal to 0.11 ± 8 × 10^−3^ (Figure 3).

The analysis of PRSs integrated into the three above-mentioned tRNA-encoding genes showed that Phs similar to phage RR1-A were predominantly integrated into the gene encoding tRNA^Thr^(GGU)_010_. In addition, phiLM21-like int-Phs were significantly more frequently integrated into the gene encoding tRNA^Lys^(CUU)_039_ (X^2^ = 15.95, P = 2.7 × 10^−3^), but similar sequences were also detected in gene 016. GIs were significantly more frequently used for integration into the sequences of the gene encoding tRNA^Asn^(GUU)_030_ (X^2^ = 12.8, P = 1.2 × 10^−3^). GIs were more frequently integrated than Phs in genes 008, 013, and 010. The occurrence of repeated integration into the same gene, namely, gene 039, supports the conclusion that integration “hot spots” are favored. Thus, in the chromosome of strain KH46, a 137.5 kb sequence was detected as integrated into the gene encoding tRNA^Lys^(CUU)_039_, which is flanked by 49 bp direct repeat (DR) identical to the 3′ end of the gene. There are 46 bp identical to the flanking DR just in the middle of this sequence. Apparently, the 137.5 kb sequence was formed by the sequential integration of a GI and then an int-Ph, whose putative sizes were apparently 84.2 and 53.3 kb, according to PHASTER and PHASTEST annotation (Table S2).

In conclusion, tRNA genes 010, 030, and 039 are proposed to be sites of preferential integration of PRSs, or integration “hot spots”, on the *Sinorhizobium meliloti* chromosome (the preferential insertion of GIs and Phs into corresponding tRNA gene sequences is a statistically reliable fact).

### 2.3. tRNA Genes in PRSs (tRNA_PRS_)

Twenty-three tRNA genes were identified in 22 PRSs integrated into the sequences of tRNA genes in 13 out of the 27 studied *S. meliloti* strains (Table 4). These tRNA genes (hereafter, tRNA_PRS_) were in addition to the tRNA genes located on the chromosomes of the strains studied. Two tRNA genes were identified in two PRSs that were also located on the chromosome but were not integrated into tRNA genes (Table 4). A total of 25 tRNA_PRS_ genes encoded the following tRNAs: nine tRNAs ^fMet^(CAT), eight tRNAs ^Met^(CAT), four tRNAs ^Thr^(GGT), two tRNAs ^Leu^(TAG), and one tRNA each for ^Val^(CAC) and ^Ser^(GCT).

As a result, we showed for the first time that PRSs can contain one or two genes encoding tRNAs at the same frequency of 0.38. Cases of three or four tRNA genes in PRSs are rare occurrences (two and one strain of *S. meliloti*, respectively).

tRNA_PRS_ genes were found to be 4-fold more frequent in Phs than in GIs (0.82 and 0.18, respectively). These genes could be within one or several different PRSs, similar to different rhizobiophages or phages infecting bacteria belonging to different taxa. The four tRNA_PRS_-encoding genes identified in the genome of strain M270 were represented by two genes encoding tRNA^fMet^(CAU)_PRS_ within two phiLM21-like int-Phs and two tRNA^fMet^(CAU)_PRS_-encoding genes located within inc-Phs: one was similar to *Sulfitobacter* phage NYA-2014a, and the other was similar to *Paracoccus* phage Shpa (Table 4).

The sequence similarity in the genes encoding tRNA_PRS_ and the corresponding chromosomal tRNA genes was assessed. As an example, two tRNA_PRS_-encoding genes in strain AK83 were analyzed. These are the genes encoding tRNA^Val^(CAC)_PRS_ and tRNA^Ser^(GCU)_PRS_, each located within one of two int-Phs. One was a phiLM21-like int-Ph, and the other int-Ph was similar to both *Ruegeria* phage DSS3-P1 and *Loktanella* phage pCB2051-A simultaneously. The similarity level of the gene encoding tRNA^Ser^(GCU)_PRS_ with the analogous chromosomal gene encoding tRNA^Ser^(GCU)_016_ did not exceed 96.7% (coverage 100%) but was higher with the gene encoding tRNA^Ser^(GCU) detected in the genomes of *Stappia indica* PHM037 and *Jiella pelagia* HL-NP1 (GenBank: CP046908.1 and CP114029.1, respectively; identity 98.9%; coverage 100%). For the gene encoding tRNA^Val^(CAC)_PRS_, no similar sequences were detected in the genomes of the studied *S. meliloti* strains or those of other bacterial species (coverage < 45%).

Another example is the analysis of tRNA^Leu^(UAG)_PRS_ genes present in two int-Phs similar to *Mesorhizobium* phage vB_MloP_Lo5R7ANS (strains AK21 and RU11/001, Table 4). The analysis showed that the similarity in the tRNA^Leu^(UAG)_PRS_ gene to the sequence of the corresponding gene located on the chromosome, tRNA^Leu^(UAG)_026_, did not exceed 80.6%, but it was significantly higher (97.6%) when compared with the corresponding genes detected in both *Octadecabacter arcticus* 238 (GenBank: CP003742.1) and *O. antarcticus* 307 (GenBank: CP003740.1). Thus, the gene encoding tRNA^Leu^(UAG)_026_ was inactivated by PRS integration (see above) but replaced by the tRNA^Leu^(UAG)_PRS_ gene, similar to that in another species of *Alphaproteobacteria*, as mentioned above.

The sequences of three genes encoding tRNA^Thr^(GGU)_PRS_ identified in three GIs that had inc-Phs similar to phage RR1-A (strains RU11/001, SM11, USDA1157; Table 4) were shown to be identical to each other. All three genes were identical to the gene encoding tRNA^Thr^(GGU)_PRS_ present in an RR1-A-like int-Ph integrated into gene 050 (strain USDA1021). All four sequences encoding tRNA^Thr^(GGU)_PRS_ differed from the sequence of gene 010 (coverage 59%, identity 95.8%) and had no similarity to the corresponding genes in other bacterial species.

Thus, we showed that PRSs can contribute additional tRNA genes to the host bacterial genome. Such tRNA genes have higher similarity to the corresponding genes of bacteria of other species than to the corresponding tRNA gene in the host bacterium. Such substitutions resulted in the diversification of evolutionarily old genes in host bacterial genomes and could in principle help the codon adaptation of genes harbored by GIs. The presence of identical genes encoding tRNA_PRS_ in prophages and genomic islands is evidence confirming that the origin of GIs are the corresponding prophages.

**Figure 4:**
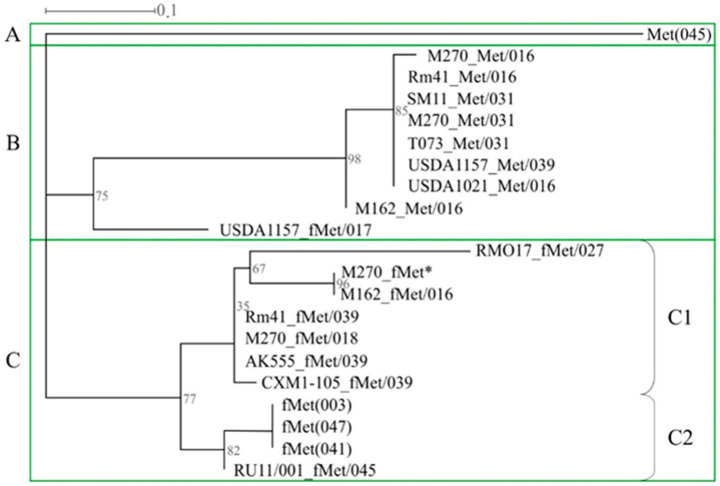
Phylogenetic analysis of genes encoding essential tRNA^Met^(CAT) and tRNA^fMet^(CAT) of *S. meliloti* strains. Designations: the signatures of tRNA genes are in the form X_Y/N, where X—the name of the strain, Y—amino acid Met or fMet, N—ordinal number of the tRNA gene in which the PRS with an additional tRNA gene is integrated; see also the designations given in Table 3; *—additional tRNA^fMet^(CAT) located in PRS that is not integrated into the tRNA gene.

Phylogenetic analysis was performed on the nucleotide sequences of genes encoding tRNA^Met^(CAU)_PRS_ and tRNA^fMet^(CAU)_PRS_, which occurred most frequently in the genomes of the strains studied (frequency of 0.68). As a result, we analyzed the sequences of 17 genes encoding tRNA^Met^(CAU)_PRS_ and tRNA^fMet^(CAU)_PRS_, as well as the sequences of genes 003, 041, and 047, belonging to the three *rrn* operons, and gene 045, located on the chromosome (Figure 4). Three clusters, A, B, and C, were identified. Cluster A had only the sequence of gene 045 and was identical in all *S. meliloti* strains studied (Figure 4). Cluster B included the sequences of eight tRNA^fMet^(CAU)_PRS_ genes belonging to phiLM21-like phages, as well as the sequence of the tRNA^fMet^(CAU)_PRS_ gene belonging to an inc-Ph similar to *Paracoccus* phage Shpa (the bootstrap value was 75%; Figure 4). The third cluster (C) comprised two monophyletic subclusters, C1 and C2 (bootstrap 77%). Subcluster C1 combined the sequences of seven tRNA^fMet^(CAU)_PRS_-encoding genes belonging to PRSs similar to different phages, including the rhizobiophages *Sinorhizobium* phage phiLM21 and *Rhizobium* phage 16-3, as well as phages Shpa and NYA-2014a, which infect *Paracoccus* and *Sulfitobacter,* respectively (Table 4). Subcluster C2 was represented by three tRNA^fMet^(CAU) genes in *rrn* operons, as well as the sequence of the tRNA^fMet^(CAU)_PRS_ gene, which was part of a Ph similar to *Rhizobium* phage 16-3 and was integrated into gene 045 (strain RU11/001; Figure 4).

In summary, our results clearly show, for the first time, that tRNA genes present in the *S. meliloti* genome have different phylogenetic origins and, apparently, are linked to horizontal transfer processes involving phages, which is also true for tRNA^fMet^(CAU)_PRS_-encoding genes, similar to those from *rrn* operons. It should be noted that there is no evidence that tRNA_PRS_ genes are used as new potential sites for integration.

### 2.4. The Synteny of PRSs Integrated into Essential tRNA Genes

**Figure 5:**
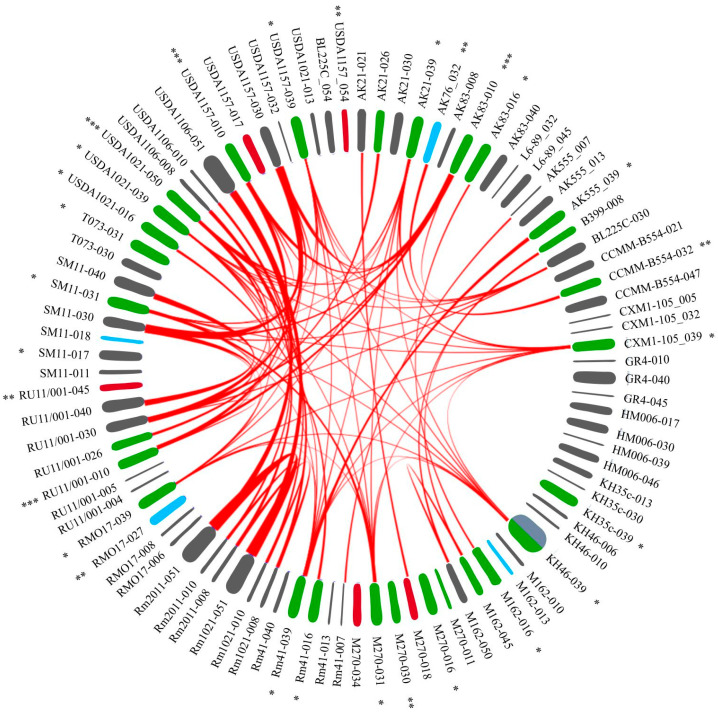
The synteny blocks of PRSs integrated into essential tRNA genes of *S. meliloti.* Designations: there are sequences of PRSs around the circle; PRS signature: the strain that contained a PRS, with the ordinal number of the tRNA gene following the dash (see Table S2, Figure 2). Type of PRS indicated by color: gray—GI, green—int-Ph, blue—q-Ph, red—inc-Ph (see text). The red lines inside the circle connect matching sequences between pairs of PRSs (see Section 4 Materials and Methods; Table S3B). Detailed blastn PRS alignment results are in Table S3A. *, **, ***—PRS similar to phages phiLM21, 16-3, and RR1-A, respectively (see text). USDA1157-054, BL225C-054, and USDA1021-013—PRSs localized on the megaplasmid pSymB.

Using pairwise sequence alignment (see Section 4 Materials and Methods), we analyzed the sequence synteny of the 88 above PRSs and the 3 PRSs that were also integrated into tRNA genes but located on pSymB (Table S2). This allowed us to identify the top-matching alignment results (hereinafter, synteny blocks or SBs) between all PRSs, amounting to 1057 SBs. SB sizes ranged from 37 to 71,338 bp (identity 73.6–100%; Table S3A), and of these, 983 were less than 8 kb (identity < 90%) and were not considered further (Figure S1). Of interest for analysis were 83 SBs, each with a size larger than 8 kb (identity > 90%; Table S3B). These SBs were identified between 19 GIs and 25 Phs, whereas the other 32 GIs and 12 Phs did not have the above SBs. There were 48 SBs between Ph/Ph pairs, 18 SBs between Ph/GI pairs, 7 SBs between GI/GI pairs that had phage-like sequences, and 10 SBs between GI/GI pairs that were free of such sequences (Figure 5). It should also be noted that the lack of detected SBs was 2.6 times more frequent in GIs than in Phs.

#### 2.4.1. Analysis of 48 SBs between Ph/Ph Pairs

Forty-six synteny blocks were detected between rhizobiophage-like Phs. Of these, 39 SBs were between phiLM21-like Phs integrated into particular tRNA genes (Table 3, Figure 2). The integration points of these PRSs were genes 016, 031, and 039. Six other SBs were between RR1-A-like Phs integrated into genes 010 and 050, and one block was between an int-Ph and a q-Ph similar to *Rhizobium* phage 16-3 integrated into gene 032 (Figure 5). In addition, a block between pairs of phages similar to *Mesorhizobium* phage vB_MloP_Lo5R7ANS (integrated into gene 026) and another block between pairs of phages similar to *Sulfitobacter* phages (integrated into genes 008 and 045) were identified (Figure 5, Table S3B).

#### 2.4.2. Analysis of 18 SBs between Ph/GI Pairs

A Ph/GI-pair analysis revealed 15 SBs between four int-Phs (integrated into genes 010 and 050) and four GIs (integrated into gene 030) containing fragments of *Rhizobium* phage vB_RleM_PPF1, and 3 SBs were identified between Ph/GI pairs that had sequences similar to those of the *Sulfitobacter* phage (integrated into genes 008, 018, 045, 021, and 050) (Figure 5; Table S3B).

#### 2.4.3. Analysis of 17 SBs between GI/GI Pairs

Seven SBs were detected between pairs of GIs that had sequences similar to phages. Of these, six blocks were between four GIs integrated into gene 030, each containing a q-Ph similar to *Rhizobium* phage vB_RleM_PPF1. In one of these GIs, the sequence of interest was identified only through its similarity to the above sequences (using BLASTn; strain USDA1157). Another SB was between pairs of GIs integrated into gene 040, and each harbored sequences similar to both *Enterobacter* phage HK542 and *Bacillus* phage BM5 (Figure 5; Table S3B).

Ten SBs were detected between pairs of GIs that did not have sequences similar to phages. Of these, nine SBs (ranging from 14.8 to 71.3 kb) were between pairs of GIs integrated into genes 008, 010, and 051, which were identified in the genomes of closely related strains (1021, 2011, and USDA1106). The GI integrated into gene 051 of strain USDA1106 had a sequence similar to that of Stx-encoding phages, which did not overlap with SBs. Another block was between pairs of GIs integrated into gene 007 (Figure 5; Table S3B).

Thus, the highest number of synteny blocks was detected between pairs of Phs, whereas the number of blocks was 2.7-fold lower between pairs of Ph/GI sequences and even less frequent, namely, 2.6-fold, between pairs of GIs (48, 18, and 7 blocks, respectively). The decrease in the number of SBs identified in the pairwise sequence comparison of these variants is similar to a decreasing geometric progression whose denominator is (2.62 ± 0.05)^−1^.

A comparative analysis of GIs and Phs showed that sequences similar to rhizobiophages were abundant in prophages, namely, in 27 out of 37 Phs (frequency 7 × 10^−1^), and were extremely rare in GIs. The sequences of interest were only in 3 out of 51 GIs, i.e., the indicated sequences were an order of magnitude less frequent in GIs (frequency 6 × 10^−2^). Thus, rhizobiophage-like sequences introduced into the genomes of *S. meliloti* strains are not maintained (not “fixed”). This appears to be the result of the cell’s defense systems against DNA of phage origin or “foreign DNA”. At the same time, in GIs, there is a “fixation” of phage sequences for which rhizobia are not specific hosts. This may contribute to the formation of bacterial defenses against superinfection, i.e., the formation of immunity to phages belonging to the same immunity group as the “fixed” prophage.

#### 2.4.4. Analysis of SBs between PRSs of *Sinorhizobium* spp.

A search for sequences similar to the rhizobiophages phiLM21, RR1-A, and 16-3 identified in *S. meliloti* strains was also carried out in the full-genome sequences of various species of the genus *Sinorhizobium* available in GenBank (access date June 2024). Extended sequences similar to *Rhizobium* phage 16-3 were not found at all in the genomes of other members of the genus *Sinorhizobium*, and these results are in agreement with the data that we obtained previously [67]. 

SBs were detected between phiLM21-like Phs present in the genomes of six *S. meliloti* strains (AK21, AK555, CXM1-105, USDA1157, RMO17, and Rm41), both strains of *S. kummerowiae*, and 13 tested *S. medicae* strains (coverage > 50%, identity > 90%; see Section 4 Materials and Methods; Table 5). The similarity in the indicated SBs was higher in the pairwise comparison of PRSs from *S. meliloti*/*S. kummerowiae* than in the pairwise comparison of PRSs from *S. meliloti*/*S. medicae* (coverage 61–78%, identity 92.90–99.54% and coverage 53–68%, identity 90.24–98.49%, respectively).

SBs similar to rhizobiophage RR1-A were found in all tested *S. medicae* strains (coverage 52–100%, identity 92.53–97.98%), except for three strains (SU277, T2, and T10) (see Section 4 Materials and Methods; Table 5). The above-indicated SBs were only in 4 out of 27 *S. meliloti* strains (USDA1157, RU11/001, AK83, and USDA1021) and were absent in both mentioned *S. kummerowiae* strains. PRSs similar to rhizobiophage RR1-A identified in *S. meliloti* strain USDA1021 and *S. medicae* strains ml42 and ml20 were found to have a high degree of similarity (coverage 100%, identity 97.98%).

Consequently, SBs similar to rhizobiophages phiLM21 and RR1-A were abundant in *S. medicae* strains (100% and 77% of strains of those tested, respectively), while the above sequences were found in *S. meliloti* strains almost 5-fold and 7-fold less frequently than in *S. medicae* strains. Both SBs similar to RR1-A- and phiLM21-like phages were identified in only one *S. meliloti* strain (USDA1157) and almost all tested *S. medicae* strains. Thus, it could be concluded that two closely related species, *S. meliloti* and *S. medicae*, as well as *S. kummerowiae*, recently assigned to *S. meliloti* species [68], are significantly distinct in terms of the infectivity of these two rhizobiophages.

### 2.5. Analysis of Integrases Identified in PRSs

**Figure 6:**
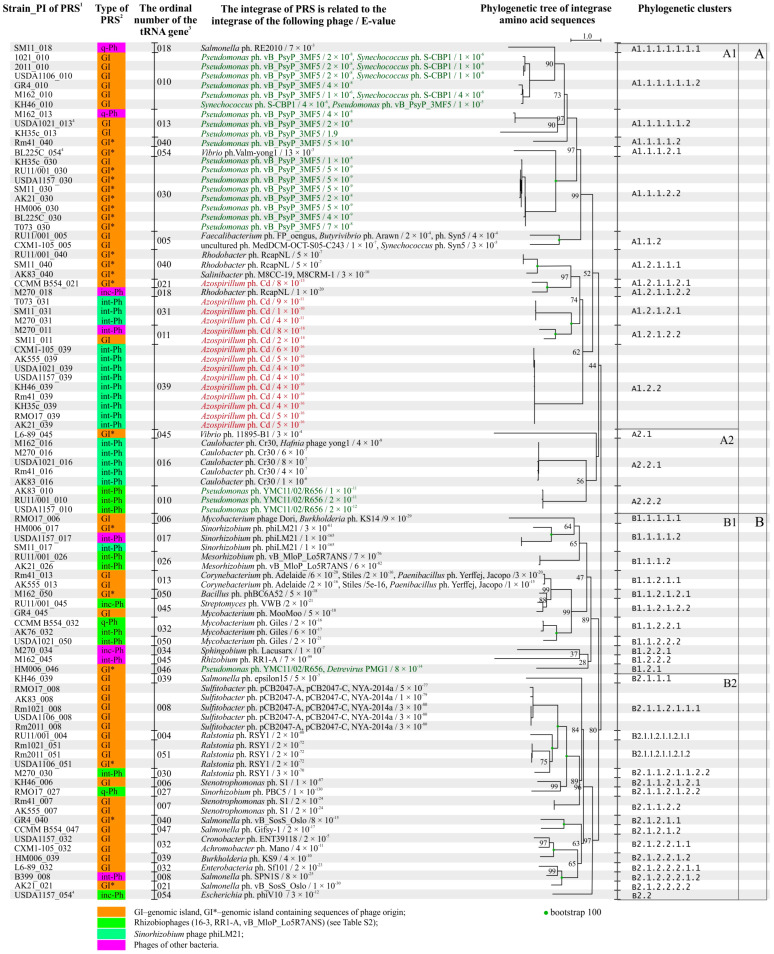
Phylogenetic tree constructed using the amino acid sequences of integrases of PRS integrated into tRNA genes of *S. meliloti*. Designations: ^1^—PI means a point of integration, it combines the strain and an ordinal number of tRNA genes through a dash (see Table 3); ^2^—GI—genomic island; int-Ph—intact phage; inc-Ph—incomplete phage sequence; q-Ph—questionable phage sequence; ^3^—ordinal numbers of tRNA genes (see Table 3); ^4^—PRS localized on pSymB in case of the corresponding strains (see text and Table S2).

All 88 PRSs (51 GI and 37 Phs) had *att* sites and were site-specifically integrated into different tRNA genes (Figure 6). All of these sequences had a gene encoding an integrase that is responsible for site-specific integration. All identified integrases were tyrosine integrases similar to phage integrases, except one showed similarity not only to the tyrosine integrase of *Mycobacterium* phage Dori but also to the serine recombinase of *Burkholderia* phage KS14 (Figure 6).

Both the nucleotide and amino acid sequences of PRS integrases shared similarities with certain phage integrases in only 14 of the 88 cases. This was the case for three *Sinorhizobium* phage phiLM21-like integrases, two *Mesorhizobium* phage vB_MloP_Lo5R7ANS-like integrases, five integrases similar to integrases of different *Sulfitobacter* phages, and four *Ralstonia* phage RSY1-like integrases (E-value ≤ 4 × 10^−61^; according to viruSITE) (Figure 6). In the other 74 cases, similarity to the integrases of different phages was established based on amino acid sequence analysis (E-value ≤ 7 × 10^−3^; in 1 case, the E-value was 1.9 (Figure 6, Table S4)). In total, similarities to the integrases of 44 different phages were detected, but half of these phages belonged to 15 different families within the class *Caudoviricetes* and their host bacteria belonged to 25 distant taxa (Table S4). The remaining phages were unclassified *Caudoviricetes*, and one of them was an unclassified bacterial virus, according to the latest phage classification [69,70]. 

The genes encoding integrases identified in each of the 51 GIs were analyzed. Only 1 of the 51 sequences was similar to a rhizobiophage integrase, namely, *Sinorhizobium* phage phiLM21. All other sequences were predominantly similar to integrases of different phages infecting *Sulfitobacter* or *Pseudomonas*, which are representatives of *Alphaproteobacteria* and *Gammaproteobacteria*, respectively (frequency of 0.61). Similarity to integrases of phages infecting *Betaproteobacteria* or bacteria of other classes was also revealed (average frequency of 0.15), while similarity to integrases of phages infecting bacteria from other genera of *Alphaproteobacteria* was extremely rare (frequency of 0.08; Table S4). A significant difference was observed between the group of GIs that did not have sequences of phage origin (except genes encoding integrases) and the group of GIs that had such sequences. This difference was evidenced by the occurrence of integrases similar to those of a larger number of different phages in the former group (Shannon indices H = 1.55 and 1.08, respectively), in which integrases of different *Sulfitobacter* phages and *Pseudomonas* phage vB_PsyP_3MF5 (Χ^2^ = 9.3, P = 2.5 × 10^−2^) were abundant (Figure 6; Table S4).

The genes encoding integrases identified in each of the 37 Phs were also analyzed. These integrases were predominantly similar to integrases of phages infecting different representatives of *Alphaproteobacteria* (frequency of 0.70), according to amino acid sequence analysis. Half of these sequences were similar to phage Cd integrase; this phage infects associative nitrogen-fixing soil bacteria of the genus *Azospirillum* (Figure 6). About 30% of the other sequences were similar to integrases of phages infecting the following bacteria of distant taxa: *Betaproteobacteria* (*Ralstonia*), *Gammaproteobacteria* (*Salmonella*, *Pseudomonas*), and *Actinomycetia* (*Mycobacterium*, *Streptomyces*) (Shannon index H = 0.88; Table S4).

It is noteworthy that the origin of the integrase (according to viruSITE) rarely matched the origin of the PRS (according to PHASTER and PHASTEST). There are only three cases, namely, a phiLM21-like Ph and two Phs similar to the *Mesorhizobium* phage vB_MloP_Lo5R7ANS, where the integrase and PRS had the same origin. In all other cases (34 of 37), where the PRS integrases were similar to integrases of other phages, according to amino acid sequence analysis. For example, in 18 phiLM21-like Phs, an integrase corresponding to the phiLM21 phage was found only once, whereas in other cases, the similarity was predominantly to the integrase of *Azospirillum* phage Cd or, with half the frequency, to the integrase of *Caulobacter* phage Cr30 (frequencies of 0.67 and 0.28, correspondingly) (Table S4).

Thus, the integrases detected in Phs and GIs (with and without sequences of phage origin) originated from different groups of phages infecting predominantly *Alpha*- and *Gamma*-/*Betaproteobacteria*, respectively, and the detected difference was significant (Χ^2^ = 20.5 and 23.7, *p* < 0.05).

Integrases similar to *Azospirillum* phage Cd and various *Pseudomonas* phages constituted the most representative groups, 15 and 21 sequences, respectively, of the 88 sequences tested (frequencies of 0.17 and 0.24, respectively). Therefore, they were used for a phylogenetic analysis conducted jointly considering PRS integration sites.

The 15 sequences of integrases similar to *Azospirillum* phage Cd integrase formed four subclusters in cluster A1.2. It turned out that the sequences of integrases that clustered in subclusters also coincided with the integration sites of the corresponding PRSs, namely, gene 039, 031, 021, or 011. The frequencies of occurrence of the integrase sequences in the corresponding subclusters were 0.60, 0.20, 0.07, and 0.13, respectively (Figure 6).

We analyzed the similarities between the amino acid sequences of PRS integrases and *Azospirillum* phage Cd integrase. Both integrase sequences had a higher degree of similarity when PRS integration occurred at the “hot spot”, gene 039 (subcluster A1.2.2 (bootstrap 62)), compared with when the PRS had integrated into another gene, such as gene 031 (subcluster A1.2.1.2.1 (bootstrap 100); E-value 4 × 10^−16^–6 × 10^−16^ and 4 × 10^−11^–1 × 10^−10^, respectively; Figure 6). In the cases where PRSs integrated into genes 021 and 011, the corresponding integrases had a lower degree of similarity to the integrase sequence of *Azospirillum* phage Cd and belonged to the following subclusters: A1.2.1.1.2.1 (bootstrap 100) and A1.2.1.2.2 (bootstrap 100; Figure 6, Table S4), respectively. Two GIs also appeared to be integrated into the same gene 039 in different strains. However, the integrase of one of the GIs was similar to that of *Burkholderia* phage KS9, and that of the second GI was similar to that of *Salmonella* phage epsilon15. These integrases belonged to different clusters (clusters B2.1.2.2.1.2 (bootstrap 100) and B2.1.1.1 (bootstrap 84)) (Figure 6).

The 21 integrase sequences similar to *Pseudomonas* phage integrases were identified in GIs (with or without phage genes) and prophages (8:9:4 ratio, respectively) and were mainly integrated into genes 010 and 030 (frequencies of 0.43 and 0.38, respectively) but were also detected in genes 013, 040, and 046 (frequencies of 0.09, 0.05, and 0.05, respectively) (Figure 6, Table S4). Out of the 21 integrase sequences, 17 belonged to four subclusters of cluster A1.1.1 (bootstrap 99) (Figure 6). Sequences grouped into subclusters also coincided with the integration sites of the corresponding PRSs (Figure 6). 

We analyzed the amino acid sequences of integrases from this group. The integrases of GIs identified in “hot spot” gene 030 had a higher degree of similarity to the *Pseudomonas* phage vB_PsyP_3MF5 integrase (subcluster A1.1.1.2.2 (bootstrap 97); E-value 4 × 10^−9^–1 × 10^−8^) compared with the sequences of integrases of GIs identified in gene 010 (subcluster A1.1.1.1.1.1.2 (bootstrap 90); E-value 4 × 10^−8^—1 × 10^−6^) (Figure 6). Moreover, five of the six integrase sequences in the last cluster were also similar to those of *Synechococcus* phage S-CBP1 integrases (Figure 6). Three int-Phs appeared to be integrated into the same gene (010), but their integrases were similar to the integrase of *Pseudomonas* YMC11/02/R656 phage, and these sequences belonged to another cluster A2.2.2 (bootstrap 56; Figure 6, Table S4). 

Therefore, “hot spot”-integrating PRSs containing integrases that show a high degree of similarity to the integrase of the corresponding original phage may have a preferential ability to insert into the host bacterial genome.

### 2.6. Analysis of PRS Attachment Sites (att)

Using two integration “hot spots”, genes 039 and 030, and gene 010, we analyzed the complementarity in the sequences of the *attP* and *attB* sites corresponding to PRSs and the sequence of the corresponding tRNA gene in *S. meliloti*. The gene sequences of the corresponding tRNAs of other bacterial species that were potential hosts for the aforementioned phage groups were used as a comparison group.

**Figure 7:**
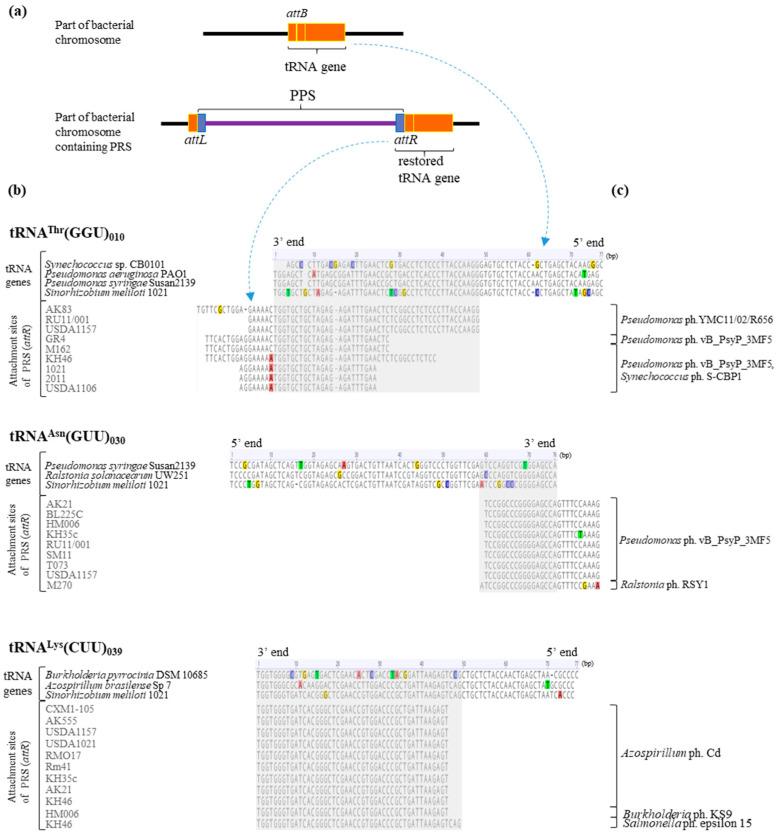
The alignment of the 3′ ends of essential tRNA genes and PRS attachment sequences (*attR* sites). Designations: (**a**)—principal scheme of PRSs integrated into tRNA genes; (**b**)—the alignment of the 3′ ends of tRNA genes of bacteria and attachment sites of PRS detected in *S. meliloti* strains; and (**c**)—phages whose integrases were similar to those of the PRSs. The similarity in phage integrases and PRS integrases was determined by analyzing their amino acid sequences (see text).

The analysis of the *attP* sites of PRSs integrated into “hot spot” gene 039 showed that these sequences were identical and 46 bp long in 10 cases, and another *attP* site was 3 bp longer (strain KH46). Both 46 and 49 bp long *attP* sites were found to be identical to *attB* sites located at the 3′ end of gene 039 in *S. meliloti* (Figure 7). 

The sequence similarity between gene 039 of *S. meliloti* 1021 and its counterparts encoding tRNA^Lys^(CUU) in potential bacteria hosts of the corresponding phages mentioned above was analyzed. The similarity to the corresponding gene in *Azospirillum brasilience* Sp7 was 94.7%, whereas the similarity to the same gene in *Burkholderia pyrrocinia* DSM 10685 was lower, at 83.1% (coverage 100% in both cases). The *attB* site corresponding to the sequence at the 3′ end of the gene encoding tRNA^Lys^(CUU) had two and nine SNPs in *Azospirillum brasilience* Sp7 and *Burkholderia pyrrocinia* DSM 10685, respectively (Figure 7). In *Salmonella enterica* LT2, a gene encoding tRNA^Lys^(CUU) was not detected at all.

The *attP* sites of PRSs integrated into “hot spot” gene 030 were 27 bp long in eight cases, and another *attP* site had one nucleotide more (Figure 7). These *attP* sites had 17 or 18 bp (respectively; see above) corresponding to the sequence at the 3′ end of gene 030 and 10 bp in the intergenic spacer adjacent to the 3′ end of the gene (Figure 7). In the case of two *attP* sites, the intergenic spacers included transitions, namely, C/T and G/A in one and A/G in the other sequences (strains KH35c and M270, respectively; Figure 7).

The sequence similarity between gene 030 in *S. meliloti* 1021 and its counterparts encoding tRNA^Asn^(GUU) in potential bacterial hosts of the corresponding phages was analyzed. The similarity to the corresponding genes in *Pseudomonas syringae* Susan2139 and *Ralstonia solanacearum* UW251 was 85.5% and 89.5%, respectively (coverage 100%), because of the presence of four SNPs in each of the two sequences (Figure 7).

The analysis of the *attP* sites of the nine PRSs integrated into gene 010 showed that their sequences varied significantly, from 31 to 64 bp. However, only 23 to 47 bp corresponded to the sequence at the 3′ end of gene 010, and the rest of the *attP* site (6 to 17 bp) was in the sequence of the intergenic spacer adjacent to the tRNA gene (Figure 7). SNPs, namely, a C/A nucleotide transversion adjacent to the tRNA gene and a G/A nucleotide transversion, were detected in the intergenic spacer regions in the case of five *attP* sites, and one other *attP* had a single-nucleotide deletion (Figure 7). Consequently, the *attP* sites of PRSs integrated into gene 010 are more variable in sequence length than the *attP* sites presented above in cases of “hot spots”.

We analyzed the sequence similarity in gene 010 in *S. meliloti* 1021 to its counterparts encoding tRNA^Thr^(GGU) in potential bacterial hosts of the above phages. The similarity did not exceed 85% (coverage from 96.1% to 100%; identity from 76.6% to 84.4%) because of the presence of SNPs in the *attB* site (Figure 7).

Thus, in the “hot spots” of integration (genes 030 and 039) and the spot of preferential integration (gene 010) in *S. meliloti*, single-nucleotide substitutions never occurred at an *att* site corresponding to the sequence at the 3′ end of a particular tRNA gene. 

Finally, it is intriguing that the *attB* sequences of the above tRNA genes in *S. meliloti* are recognized by PRS integrases that have a high level of similarity to phage integrases specific to bacteria of the genera *Azospirillum* and *Pseudomonas*.

## 3. Discussion

In this study, we conducted phage-related sequences (PRSs) analysis on the genomes of nonpathogenic, agriculturally valuable nitrogen-fixing root nodule bacteria. We focused on *Sinorhizobium meliloti* strains, which form symbiotic relationships with leguminous grasses of the genera *Medicago*, *Melilotus*, and *Trigonella.*

Our findings revealed a significant enrichment of PRSs within the genomes of these strains. The total information capacity of these “foreign DNA” sequences (314 PRSs) is comparable to the genome size of the reference *S. meliloti* 1021 strain (6.93 Mb). Surprisingly, over 64% of this “foreign DNA” is located on the chromosomes of these strains, despite chromosomes typically being considered highly conserved [71,72,73]. Therefore, we can assume that these “foreign DNA sequences” become genetic objects that are inherited through vertical evolutionary pathways, which may contribute to active processes of genetic diversification and microbial genome evolution, in agreement with [5]. 

The fact that the Phs of geographically distant *S. meliloti* strains are similar to a limited number of phages and mainly to *Sinorhizobium* phage phiLM21 was unexpected. This result leads us to suggest that *S. meliloti* has not yet properly developed defense systems against infection with this phage and that phiLM21-like phages are probably the most active participants in rhizobia lysogenization in modern ecosystems. This is confirmed by the fact that similar phiLM21-like sequences were also detected in strains of the closely related species *S. medicae*, as well as in *S. kummerowiae*.

The analysis of GIs, whose number and size varied considerably among *S. meliloti* strains, showed, for the first time, that they predominantly contained sequences similar to fragments of phages infecting bacteria of distant taxa (according to PHASTER and PHASTEST annotation). This similarity may indicate the presence of “gene flow” between phylogenetically distant bacteria involving phages, which is in agreement with [74]. In addition, the presence of such sequences in the genomes of bacterial hosts may contribute to the development of defense systems against superinfections, according to [41,75].

The analysis of *S. meliloti* GIs showed, for the first time, that there are GIs with and without sequences of phage origin, although both types of GI sequences retain genomic island features, according to the Islander algorithm [76]. Since each of the GIs studied had an integrase-encoding gene and was flanked by direct repeats, *attL* and *attR* sites, these sequences were most likely derived from a site-specific recombination mechanism similar to Lambda phage integration. The obtained data are consistent with the previously proposed theory of the “life cycle” of GIs [4], as well as with the theory of the “grounding” of prophages in the bacterial genome [5]. However, the presented data also demonstrate an active “washout” of phage genes from bacterial genomes, which is apparently associated with the action of host bacterial defense systems.

Of the identified PRSs, 28% were inserted into chromosomal tRNA genes, and these sequences were of most significance for this analysis. Of great interest is the finding that these integrated sequences represented more than half of the total amount of foreign DNA (53.5%) detected in the studied *S. meliloti* strains. Here, for the first time, we present data showing that PRS integration does not occur randomly and does not affect all tRNA genes located on the chromosome, but only half of the 52 tRNA genes identified in the genome of *S. meliloti* species. The *attB* sites of the three genes encoding the isoacceptor tRNA^Thr^(GGU), tRNA^Asn^(GUU), and tRNA^Lys^(CUU) are proposed to be “hot spots (sites)” of integration. The fact that phage sequences integrate into certain tRNAs is confirmed by recently obtained data for *Helicobacter pylori* phages [77].

The most curious and still poorly described phenomenon is that the tRNA gene that is inactivated through PRS integration is replaced by a tRNA gene that is a part of the PRS but encodes a similar isoacceptor tRNA. However, genes encoding tRNAs introduced by PRSs have greater similarity to similar genes present in the genomes of other phylogenetically unrelated *Alphaproteobacteria* species. This result suggests that a tRNA gene specific to the host bacterium is substituted by a tRNA gene that is similar to genes in taxonomically unrelated bacterial species. Such cases do not seem to be unique: a similar case was recently described for *Mycobacterium*, and the authors described it as tRNA-dependent lysogeny [49]. Therefore, there is a process for the diversification of tRNA genes, which are assumed to be conserved and evolutionarily ancient.

Thus, as a result of phage infection, bacterial genomes can acquire an additional tRNA gene, which can lead to an increase in the number of tRNAs per genome, as shown in our study. The genes encoding tRNA^fMet^ and tRNA^Met^, which are required for efficient protein synthesis in most groups of bacteria, were the most frequently introduced into *S. meliloti* genomes by PRSs. The resulting increase in the number of tRNA genes per genome may have a positive effect on the metabolic characteristics of bacteria, according to [78,79,80]. At the same time, phage-encoded tRNA was found to replace the depleted bacterial host tRNA, which occurs, for example, via the action of a specific bacterial anticodon tRNA-nuclease as an early response to phage infection [81]. Thus, the presence of phage genes encoding tRNAs may be significant in resisting bacterial antiphage defense systems and thus assist in preventing the inhibition of phage propagation [81,82,83].

All site-specifically integrated PRSs harbored a gene encoding an integrase similar to the tyrosine integrase of a phage. The fact that PRS integrases were not similar to any integrases of different rhizobiophages (viruSITE database), except in rare cases, was entirely unexpected. Notably, GIs and Phs contain integrases of different phylogenetically distant origins, a distinction we showed to be reliable. 

GIs preferentially contained integrases similar to those of *Pseudomonas* phages, whereas Phs most often carried integrases of *Azospirillum* phages. Moreover, PRSs carrying integrases of *Azospirillum* or *Pseudomonas* phages contained *att* sites identical to fragments of tRNA genes in *S. meliloti*. However, these *att* sites were not similar to the corresponding sites of tRNA genes in bacteria that were potential hosts of the corresponding phages. Thus, the results presented testify to the fact of the targeted and obviously effective insertion of “foreign sequences” into the genome of a typical nonpathogenic saprophytic soil bacterium recipient, *S. meliloti*. 

Our findings provide valuable insights into the molecular genetic mechanisms underlying phage integration into bacterial genomes and may contribute to the development of new methodologies for gene construct creation and targeted insertion vectors. Additionally, these results shed light on the role of phages in the evolution of bacteria, particularly phylogenetically distant classes like *Gammaproteobacteria* and *Alphaproteobacteria*.

## 4. Materials and Methods

### 4.1. Bacterial Genomes Analyzed in This Study

Complete genomes of 27 *Sinorhizobium meliloti* strains, 13 *S. medicae* strains, and 2 *S. kummerowiae* strains, as well as 10 strains of other genera, deposited in GenBank (accessed on 1 June 2024), were used in this study. The list of the genomes is presented in Table 5.

### 4.2. List and Purpose of the Programs Used in This Study

A search for phage-related sequences (PRSs). PHASTER (accessed on 25 June 2023), PHASTEST (accessed on 13 May 2024) [13,14,84] and Islander tools from IslandViewer4 (https://www.pathogenomics.sfu.ca/islandviewer; accessed on 5 June 2023) [53,76] were applied, respectively, to search prophages (Phs) and genomic islands (GIs), i.e., PRSs. The Islander algorithm was also used to identify the tRNA genes into which the GI integration occurred, as well as the identification of direct repeats flanking the GIs. In the case of Phs, the search for such tRNA genes was performed by aligning the flanking Ph sequences and sequences of the tRNA gene of *S. meliloti* using the BLASTn tool (https://blast.ncbi.nlm.nih.gov/Blast.cgi; accessed on 10 January 2024) [62,63].

The search and annotation of tRNA genes were carried out using Aragorn [85] and tRNAscan-SE v. 2.0 tools (https://trna.ucsc.edu/tRNAscan-SE/; accessed on 8 May 2024), which allowed us to identify the tRNA isotype and its anticodon [86,87]. The localization of tRNA genes in the chromosomes of *S. meliloti* strains was determined relative to *oriC* and *terC*.

Analysis of the structural organization of chromosomes. Sequences corresponding to the origin of replication (*oriC*) were carried out by similarity to the *oriC* (*SMc04880*) of the reference strain *S. meliloti* 1021 (477 bp) according to [59] using BLASTn [62,63]. DnaSP v. 5.10.01 software (University of Barcelona, Barcelona, Spain) [88] was used to determine nucleotide diversity index (π) for *oriC* sequences. The first nucleotide of the sequence of the *oriC* was assumed as an origin (start) point of each of the studied chromosomes. The method of standardization of coordinates of chromosomal sequences of *S. meliloti* relative to *oriC* was described in [62,63]. The origin point was assumed to be 0°, and the opposite was 360°, consistent with [89]. Coordinates in degrees were determined by the following formula: C° = Cbp/(Lbp/360), where C° is the coordinate in degrees, Cbp is the coordinate in bp, and Lbp is the length of the chromosome in bp. The potential terminus location (*terC*) was determined using a method based on an asymmetrically distributed octomeric sequence (GGGCAGGG) on both strands in the genome of the *Alphaproteobacteria*. The abundance of this octomeric sequence on each strand around the *terC* region is maximal [90]. This method is specific for *Alphaproteobacteria* and does not depend on the settings of the octomeric sequence search program. The localization of the octomeric sequence in *S. meliloti* chromosomes was determined using BLASTn. To verify the results obtained, the classical method of determining the potential *terC* sequence based on GC-skew was used. GC-skew was determined using GenSkew (https://genskew.csb.univie.ac.at/; accessed on 2 November 2023) [91]; the Stepsize and Windowsize were equal to 0.1% of the chromosome length.

The analysis of the synteny of the nucleotide sequences of PRSs was carried out using the BLASTn tool. Based on the pairwise alignment of all sequences, a table that contained the results of only the best of all the resulting alignments was created (Table S3A; Figure S1). Using the Python JSON library, this table was converted into a document with data in JSON format. A graphical representation of the obtained synteny data was obtained using the dash bio module (https://dash.plotly.com/dash-bio; accessed on 23 October 2023). For the graphic image, only the results of an alignment of more than 8 kb with a length and identity of more than 90% were used.

The viruSITE tool (accessed on 15 March 2024) [92] was used to analyze amino acid and nucleotide sequence encoded integrases and to determine the degree of similarity between integrase sequences of different phages. 

Phylogenetic trees were reconstructed using MUSCLE (https://www.ebi.ac.uk/jdispatcher/msa/muscle; accessed on 15 June 2024) [93] and IQTree tools (http://iqtree.cibiv.univie.ac.at/; accessed on 15 June 2024) [94].

The comparison analysis of different groups was performed according to the Chi-square test (significance level, α = 0.05) using PAST v. 4.03 software (Oslo, Norway) [95].

## Figures and Tables

**Table 1:** Evaluation of the occurrence of phage-related sequences (PRSs) in genomes (gray squares) and on chromosomes (white squares) in *S. meliloti* strains.

PRS *	Number of PRS Integrated into **								Total Number of PRS ***	
	Essential tRNA Gene				Protein-Coding Sequence or Nonessential Region					
	*att*+		*att*−		*att*+		*att*−		on Chrs	in Genomes
	Total	on Chrs	Total	on Chrs	Total	on Chrs	Total	on Chrs		
GI	54	51	0	0	0	0	0	0	51	54
int-Ph	30	30	0	0	11	2	12	3	35	53
inc-Ph	1	1	0	0	48	29	115	7	37	164
q-Ph	6	6	0	0	13	1	24	2	9	43
In total:	**91**	**88**	**0**	**0**	**72**	**32**	**151**	**12**	**132**	**314**
Total number in genomes ***	**91**				**223**					
Total number on chrs ***	**88**				**44**					

*—PRS: GI—genomic island; int-Ph—intact prophage sequence; inc-Ph—incomplete prophage sequence; q-Ph—questionable prophage sequence; ** *att*+/*att*−—*att* site is present or absent, respectively; attachment site were detected using PHASTER, PHASTEST and/or Islander; ***—in the 27 *S. meliloti* strains; chrs—on chromosomes of studied strains; in bold—total number of PRS.

**Table 2:** Similarity of prophages integrated into tRNA genes of *S. meliloti* to particular phages.

PRSs Similarities to the following Phage ^1^		PRSs ^2^:			in Total:
		int-Ph	inc-Ph	q-Ph	
*Sinorhizobium* phage	phiLM21	18	0	0	18
*Rhizobium* phage	16-3	2	1	2	5
	RR1-A	4	0	0	4
*Mesorhizobium* phage	vB_MloP_Lo5R7ANS	2	0	0	2
*Brucella* phage	BiPBO1	1	0	0	1
*Sulfitobacter* phage	pCB2047-A	2	0	0	3
	NYA-2014a	0	3	0	3
*Enterobacteria* phage	mEp235	0	0	2	2
In total **PRSs**		29	4	0	37

^1^—the similarity to particular phages is according to PHASTER and PHASTEST, see NCBI RefSeq in Table S2; ^2^—Prophages identification according to PHASTER and PHASTEST: int-Ph—intact prophage; inc-Ph—incomplete prophage sequence; q-Ph—questionable prophage sequence.

**Table 3:** Essential tRNA genes of the reference strain *S. meliloti* 1021.

tRNA Isotype ^1^	Ordinal Number of tRNA Gene in Chromosome	Anticodon	tRNA Isotype	Ordinal Number of tRNA Gene in Chromosome	Anticodon
tRNA^Ala^	028	CGC	tRNA^Gln^	**013**	CUG
	**005**	GGC		014	UUG
	002, 042, 048 ^2^	UGC	tRNA^Lys^	**039**	CUU
tRNAf^Met^	003, 041, **047** ^2^	CAU		**032**	UUU
tRNA^Met^	**045**	CAU	tRNA^Val^	**046**	GAC
tRNA^Ile^	**040** ^3^	CAU		**018**	UAC
	001, 043, 049 ^2^	GAU	tRNA^Gly^	009	CCC
tRNA^Leu^	044	CAA		052	GCC
	**006**	CAG		023	UCC
	025	GAG	tRNA^Arg^	**007**	ACG
	**031**	UAA		**050**	CCU
	**026**	UAG		033	UCU
tRNA^Glu^	037	CUC	tRNA^Pro^	015	CGG
	035, 036, 038	UUC		**017**	GGG
tRNA^Ser^	**051**	CGA		**034**	UGG
	**016**	GCU	tRNA^Asn^	**030**	GUU
	**004**	GGA	tRNA^Cys^	029	GCA
	**027**	UGA	tRNA^His^	012	GUG
tRNA^Thr^	**008**	CGU	tRNA^Phe^	**011**	GAA
	**010**	GGU	tRNA^Trp^	024	CCA
	**021**	UGU	tRNA^Tyr^	022	GUA
tRNA^Asp^	019, 020	GUC	**Total: 20**	**52**	**42**

^1^—the tRNA isotype predicted according to [64]; ^2^—tRNA gene belonging to *rrn*-*rrl* operons; ^3^—isoleucine tRNA with CAU anticodon (according to tRNAscan-SE); in bold—ordinal number of the tRNA gene in which PRS is integrated at least in one strain.

**Table 4:** tRNA genes in PRSs in *S. meliloti* strains.

tRNA Gene(s) in PRS ^1^	PRS ^2^	Characteristics of PRSs			*S. meliloti* Strain
		tRNA Gene ^3^	Similarity to Phage ^4^	Sequences Similar to ^5^	
^Leu^(UAG)	int-Ph	026	*Mesorhizobium* phage vB_MloP_Lo5R7ANS ^6^	-	AK21
^Val^(CAC)	int-Ph	016	*Sinorhizobium* phage phiLM21	-	AK83
^Ser^(GCU)	int-Ph	-	*Ruegeria* phage DSS3-P1, *Loktanella* phage pCB2051-A	-	
^fMet^(CAU)	int-Ph	039	*Sinorhizobium* phage phiLM21	-	AK555
^fMet^(CAU)	int-Ph	039	*Sinorhizobium* phage phiLM21	-	CXM1-105
^Met^(CAU)	int-Ph	031	*Sinorhizobium* phage phiLM21	-	T073
^fMet^(CAU)	int-Ph	016	*Sinorhizobium* phage phiLM21	-	M162
^Met^(CAU)					
^fMet^(CAU)	q-Ph	017	*Sulfitobacter* phage NYA-2014a	-	USDA1157
^Thr^(GGU)	GI	030	-	*Rhizobium* phage vB_RleM_PPF1, q-Ph	
^Met^(CAU)	int-Ph	039	*Sinorhizobium* phage phiLM21	-	
^fMet^(CAU)	q-Ph	027	*Rhizobium* phage 16-3	-	RMO17
^Met^(CAU)	int-Ph	016	*Sinorhizobium* phage phiLM21	-	Rm41
^fMet^(CAU)	int-Ph	039	*Sinorhizobium* phage phiLM21	-	
^Leu^(UAG)	int-Ph	026	*Mesorhizobium* phage vB_MloP_Lo5R7ANS	-	RU11/001
^Thr^(GGU)	GI	030	-	*Rhizobium* phage vB_RleM_PPF1, q-Ph	
^fMet^(CAU)	int-Ph	045	*Rhizobium* phage 16-3	-	
^Met^(CAU)	int-Ph	016	*Sinorhizobium* phage phiLM21	-	USDA1021
^Thr^(GGU)	int-Ph	050	*Rhizobium* phage RR1-A	-	
^Met^(CAU)	int-Ph	016	*Sinorhizobium* phage phiLM21	-	M270
^fMet^(CAU)	inc-Ph	018	*Sulfitobacter* phage NYA-2014a	-	
^Met^(CAU)	int-Ph	031	*Sinorhizobium* phage phiLM21	-	
^fMet^(CAU)	inc-Ph	-	*Paracoccus* phage Shpa	-	
^Thr^(GGU)	GI	030	-	*Rhizobium* phage vB_RleM_PPF1, q-Ph	SM11
^Met^(CAU)	int-Ph	031	*Sinorhizobium* phage phiLM21	-	

^1^—the tRNA isotype predicted according to tRNAscan-SE; ^2^—phage-related sequences (PRSs) represented by GI—genomic island and Ph—prophage (int—intact Ph/int-Ph, q—questionable Ph/q-Ph, inc—incomplete Ph/inc-Ph); ^3^—tRNA gene (see Table 3, Figure 2); ^4^—the similarity in prophages to one or more phages according to PHASTER and PHASTEST; ^5^—the presence of sequence in GI related to one or more inc-Ph/q-Ph according to PHASTER and PHASTEST (NCBI Reference Sequence); ^6^—NCBI Reference Sequence, see Table S2.

**Table 5:** List of analyzed bacterial genomes.

Strain	NCBI BioSample	Strain	NCBI BioSample	Strain	NCBI BioSample
*Sinorhizobium meliloti*					
AK21 ^I^	SAMN08428886	HM006	SAMN07175160	RMO17	SAMN02952139
AK83 ^I^	SAMN00017059	KH35c	SAMN07175161	GR4	SAMN02603224
L6-AK89 ^I^	SAMN22420025 ^II^	KH46	SAMN07175162	CCMM B554 (FSM-MA)	SAMN06284128
AK76 ^I^	SAMN17104055 ^III^	USDA1021	SAMN07175167	Rm41	SAMEA2272434
AK170 ^I^	SAMN10256575 ^IV^	USDA1157	SAMN07175169	T073	SAMN07175166
AK555 ^I^	SAMN08826593 ^IV^	USDA1106	SAMN07175168	M270	SAMN07175164
CXM1-105 ^I^	SAMN08826592 ^IV^	1021	SAMEA3283068	BL225C	SAMN00017103
B399	SAMN06229775	2011	SAMN02603522	RU11/001	SAMEA3146337
B401	SAMN06227501	M162	SAMN07175163	SM11	SAMN02603056
*Sinorhizobium medicae*					
ml49	SAMN38634768	WSM1115	SAMN23416899	ml4	SAMN38634762
ml2	SAMN38634765	ml60	SAMN38634769	ml18	SAMN38634763
SU277	SAMN30617559	WSM419	SAMN02598363	ml29	SAMN38634766
ml42	SAMN38634767	ml20	SAMN38634754	T2	SAMN18332160
T10	SAMN18332696				
*Sinorhizobium kummerowiae*/*S. meliloti* ^V^				*Stappia indica*	
CIP 108026	SAMN38748741	CCBAU 71714	SAMN33342371	PHM037	SAMN13341662
*Pseudomonas syringae*		*Pseudomonas aeruginosa*		*Ralstonia solanacearum*	
Susan2139	SAMN19068034	PAO1	SAMN02603714	UW251	SAMN22612269
*Azospirillum brasilience*		*Burkholderia pyrrocinia*		*Salmonella enterica*	
Sp7	SAMN03159499	DSM 10685	SAMN03651233	LT2	SAMN02604315
*Jiella pelagia*		*Octadecabacter arcticus*		*Octadecabacter antarcticus*	
HL-NP1	SAMN31329523	238	SAMN02603620	307	SAMN02603621

^I^—*S. meliloti* strains isolated from an alfalfa introgressive hybridization center (Mugodzhary, NW Kazakhstan); ^II^—*S. meliloti* strains whose genomes were sequenced (MiSeq, Illumina, San Diego, CA, USA and MinION, Oxford Nanopore Technologies, Oxford, United Kingdom), assembled, and annotated with the support of WCRC “AgriTechnologies for the Future” No. 075-15-2022-320 dated 20.04.2022; ^III^ and ^IV^—strains whose genomes were sequenced (MiSeq, Illumina, San Diego, CA, USA and MinION, Oxford Nanopore Technologies, Oxford, United Kingdom), assembled and annotated under the RSF projects 20-16-00105 and 17-16-01095, respectively; ^V^—species name is given according to [68].

## Data Availability

The original contributions presented in this study are included in this article/Supplementary Material. Further inquiries can be directed to the corresponding author/s.

## Supplementary Materials

The following supporting information can be downloaded at https://www.mdpi.com/article/10.3390/ijms251910421/s1.
