# Supplementary material for: Hot Spots of Site-Specific Integration into the Sinorhizobium meliloti Chromosome
Source: Int J Mol Sci. 2024 Sep 27;25(19):10421. doi: 10.3390/ijms251910421 (PMC11476347; doi:10.3390/ijms251910421)
Supplement: Supplementary file 1 [file ijms-25-10421-s001.zip › Figure S1.pdf]

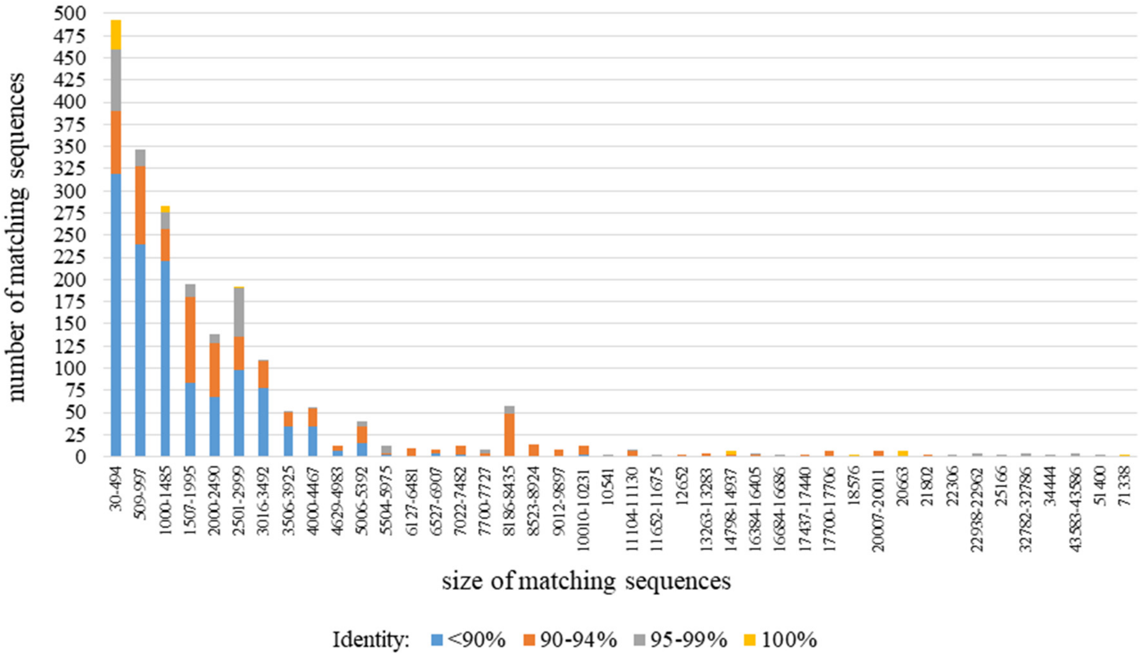

Figure S1. Number and identity (%) of matched sequences between 91 PRSs integrated into tRNA genes of *S. meliloti*. Matched sequences were identified by pairwise alignment of 91 PRSs using BLASTn (see materials and methods).
