# Supplementary material for: Hot Spots of Site-Specific Integration into the Sinorhizobium meliloti Chromosome
Source: Int J Mol Sci. 2024 Sep 27;25(19):10421. doi: 10.3390/ijms251910421 (PMC11476347; doi:10.3390/ijms251910421)
Supplement: Supplementary file 1 [file ijms-25-10421-s001.zip › Table S1.pdf]

Table S1. Estimation of the amount of genetic information contained in PRSs ("foreign DNA") within genomes (gray squares) and chromosomes (white squares) in *S. meliloti* strains (in %)*.

PRS**	The amount of PRSs integrated sequences								% on chrs****	% in genomes****
	**into essential tRNA gene *****				**into a protein-coding sequence or nonessential region *****					
	***att*+**		***att-***		***att*+**		***att-***			
	**total**	**total on chrs**	**total**	**total on chrs**	**total**	**total on chrs**	**total**	**total on chrs**		
GI	27.4	26.6	0	0	0.0	0.0	0.0	0.0	26.6	27.4
int-Ph	23.0	23.0	0	0	6.8	1.6	2.9	1.0	25.7	32.8
inc-Ph	1.1	0.6	0	0	12.7	6.4	15.2	1.3	8.3	29.0
q-Ph	3.3	3.3	0	0	4.1	0.4	3.5	0.6	4.3	10.8
In total:	54.8	53.5	0	0	23.5	8.5	21.7	2.8	64.8	100.0
% in genomes****:	**54.8**				**45.2**				**64.8**	**100.0**
% in chrs****:	**53.5**				**11.3**					

* – the sum of PRS lengths in kb is taken as the total amount of "foreign" DNA, which is equivalent to 100%. ** – PRS: GI – genomic island; int-Ph – intact phage; inc-Ph – incomplete phage sequence; q-Ph – questionable phage sequence; *** *att* +/*att-* – *att* site is present or absent, respectively; attachment site were detected using PHASTER and/or IslandViewer-4; ****– in the 27 *S. meliloti* strains; chrs – chromosomes.
